# The Tentacular Spectacular: Evolution of Regeneration in Sea Anemones

**DOI:** 10.3390/genes12071072

**Published:** 2021-07-14

**Authors:** Chloé A. van der Burg, Peter J. Prentis

**Affiliations:** Faculty of Science, School of Biology and Environmental Science, Queensland University of Technology, Brisbane 4000, Australia; p.prentis@qut.edu.au

**Keywords:** Actiniaria, regeneration, molecular genomics, innate immune system, development, evolution

## Abstract

Sea anemones vary immensely in life history strategies, environmental niches and their ability to regenerate. While the sea anemone *Nematostella vectensis* is the starlet of many key regeneration studies, recent work is emerging on the diverse regeneration strategies employed by other sea anemones. This manuscript will explore current molecular mechanisms of regeneration employed by non-model sea anemones *Exaiptasia diaphana* (an emerging model species for coral symbiosis studies) and *Calliactis polypus* (a less well-studied species) and examine how these species compare to the model sea anemone *N. vectensis*. We summarize the field of regeneration within sea anemones, within the greater context of phylum Cnidaria and in other invertebrate models of regeneration. We also address the current knowledge on two key systems that may be implemented in regeneration: the innate immune system and developmental pathways, including future aspects of work and current limitations.

## 1. Introduction

Cnidarians (jellyfish, corals, sea anemones and hydrozoans) are an ancient extant group, with estimates placing the phylum’s emergence at 740 million years ago (mya) and their divergence from its sister-lineage Bilateria at approximately 635–542 mya [1,2,3]. Despite having diverged from vertebrates several hundred million years ago, cnidarians possess surprisingly complex and vertebrate-like genomes and many gene families are highly conserved in this phylum and across metazoans [4,5,6,7,8]. Cnidarians have featured prominently among regeneration studies, with some of the earliest model species for regeneration studies found in this phylum. In fact, *Hydra* (Cnidaria: Medusozoa: Hydrozoa) is likely one of the first and oldest regeneration models, dating back as far as 1744 [9]. *Hydra* and *Nematostella* (Cnidaria: Anthozoa: Actiniaria) are currently the two main cnidarian representatives for regeneration studies and for a number of other experimental areas, such as the interaction of microorganisms with host epithelial cells [10,11], understanding evo-devo mechanisms [12,13,14,15] and exploring the evolution of the innate immune system [16,17,18,19,20].

Sea anemones (Cnidaria: Anthozoa: Actiniaria) encompass approximately 1200 species and are soft-bodied, mostly sedentary, primarily marine-dwelling invertebrates [21]. They are an evolutionary ancient extant group, with estimates placing the divergence of the model sea anemone *Nematostella* (family Edwardsiidae), from other actiniarians between 400–600 mya [22]. This is more than the estimated divergence time between mammals and birds (~326 mya) [23] and is in fact, more similar to the divergence of vertebrates (~550 mya) [24]. Considering the evolutionary distance between species within Actiniaria, it is not unsurprising that sea anemones vary immensely in life history strategies, environmental niches and their ability to regenerate.

In the wild, cnidarians, including sea anemones, are frequently preyed upon by animals such as nudibranchs, sea stars, vertebrates, and various crustaceans [25,26]. Sea anemones exhibit multiple defensive strategies to avoid or deter predators, including behavioral (e.g., moving or swimming away) [27,28] and envenomation of predators, through the firing of venom-filled cnidocytes which are distributed throughout their body plan [29,30,31]. These strategies do not always entirely deter predators, in fact, several predator species (e.g., nudibranchs) have evolved the ability to ‘steal’ cnidocytes through ingestion of cnidarian species and can repurpose these cells for their own defense [25,32]. As such, partial tissue loss through predation events on sea anemones can occur frequently in the wild, following which sea anemones must regenerate lost tissues.

Relatively few molecular or genomic studies currently exist that explore the regenerative ability of sea anemones; however, it is well-documented that some members of phylum Cnidaria, including sea anemones, have remarkable regenerative capabilities. The most infamous example is the sea anemone *Exaiptasia diaphana* (frequently referred to simply as ‘Aiptasia’, and previously named *Exaiptasia pallida* [33]). *Exaiptasia diaphana* has earned its nefarious reputation among hobbyists as an aquarium pest principally for its uncanny ability to proliferate uncontrollably. This is due both to its ability to reproduce spontaneously through pedal laceration [34,35], and to regenerate many individuals from fragmented tissue, often left behind when attempting to remove an individual from coral rock. Other sea anemones have not earned such a pestiferous status; however, many sea anemone species can also regenerate to different degrees.

Studies may use observational, morphological, cellular, molecular and genomic approaches to unravel the mechanisms underpinning regeneration. Here, we will focus on the literature exploring molecular and genomic strategies for regeneration. While there are too many cnidarian species to comprehensively review for which species regeneration has been characterized to any extent, the list for sea anemones is substantially smaller. In particular, much of the older literature described (either through visual observation or tissue sectioning/cell staining) multiple mechanisms and modes of reproduction, regeneration and unusual developmental strategies utilized by sea anemones (summarized by review [36]). Outside of the model species *Nematostella* (for which many studies exist, see review [37]), descriptions of regeneration in sea anemones include the species *Anthopleura elegantissima* [38], *Anthopleura stellula* [39], *Calliactis parasitica* [40], *Diadumene lineata* [41], *E. diaphana* [42,43,44], *Harenactis attenuata* [45], *Metridium* sp. [46], and several species in the family Boloceroididae [47].

In this review, we summarize the current knowledge of the molecular and genomic mechanisms of regeneration employed by sea anemones, as well as the current understanding of the broader mechanisms and modes of regeneration in Cnidaria. Particularly, we focus on the evolution and expression of genes involved in regeneration and how old and new genes might be utilized and co-opted from different processes. While there is still much to understand in the field of regenerative biology, insights gleaned from major models of regeneration in Cnidaria and other invertebrates can be used to form a general genetic framework for understanding whole body regeneration. Here, we also explore the role of two major systems that many studies speculate may be implemented in regeneration: the innate immune system and developmental pathways.

## 2. What Is Regeneration?

Regeneration is loosely defined as the ability to regrow or restore large portions of adult tissue, such as limbs or organs. It is not a universal trait among animal taxa; individuals may show variation in regenerative ability both temporally and ontogenically and closely related species may be unable to regenerate to the same extent [15,48,49,50,51,52]. Whether regeneration is an ancestral, conserved trait is a major question in evolutionary biology. That is, do all organisms possess a pathway, process, cellular mechanism or genetic signature that is commonly induced during regeneration? Can regeneration be considered a single evolutionarily conserved trait? This topic has been well-explored in a review by Tiozzo and Copley (2015), wherein the authors suggest considering regeneration in terms of an evo-devo approach, similar to the conceptual approaches used to understand evolutionary developmental biology. Seemingly homologous traits, such as the shared ability to regenerate limbs or a common utilization of blastema formation across species, are not necessarily evolutionarily informative when investigating regeneration [15].

Broadly, three key chronological events occur during tissue repair and regeneration in all organisms, which are: 1. Wound healing; 2. Cell population mobilization; and 3. Tissue morphogenesis. Each of these steps may be achieved in ways unique to any one particular taxon [15,48,51,52,53,54]. The same outcome may be achieved with different underlying mechanisms. Wound closure may proceed with or without cell proliferation and new tissue may be formed from stem cells, or through the dedifferentiation or transdifferentiation of somatic or progenitor cells [52]. In vertebrates such as salamanders, axolotls, and zebrafish, the primary mechanism to mobilize precursor cells during the second step of regeneration is through blastema formation [55,56]. Blastema formation at the wound site is typically driven by proliferation of resident cells or de-differentiation of lineage-committed stem cells in limb and fin models of regeneration in vertebrates [52,56,57]. In cnidarians and planarians; however, blastemas (or blastema-like proliferative zones) may be populated by migratory (rather than resident) stem cells [58,59]. Some species, such as the ctenophore *Mnemipsis leidyi,* do not produce a blastema during regeneration [60]. Here, we will focus primarily on molecular mechanisms and modes of whole-body regeneration (WBR), which pertains to regeneration of an entire individual from a very small piece of tissue or following complete dissection of an individual (either longitudinally or transversely) into two halves.

### Genetic Framework of Regeneration: Key Players Identified from Model Organisms

There are some key pathways frequently identified as being crucial in regeneratively competent organisms. The MAPK/Erk signaling pathway (functions in many processes such as cell proliferation/differentiation/survival and calcium transport modulation [61]), appears to be a prominent feature of wound healing and early regeneration across taxa. In particular, early expression of this pathway is found in almost all regenerative datasets from planarians, cnidarians (*Hydra* and *Nematostella*), sea stars, zebrafish and mice, and some evidence indicates that MAPK signaling may be required as an early inflammatory trigger to initiate or precede regeneration [62,63,64,65,66,67].

Wnt/ß-catenin and Wnt pathway components also appear to be ubiquitous in regeneration studies throughout Metazoa (incl. Planaria, Cnidaria, *Xenopus* and mammals), although this is not particularly surprising given this pathway has a well-documented role in body axis positioning and repolarization [50,68,69,70,71,72,73,74]. Interestingly, apoptotic cells have been identified as necessary providers of Wnt signaling in some species [68,75]. Other genes and pathways that are often expressed during tissue repair and regeneration include many kinds of transcription factors (e.g., NF-κB, Forkhead, RUNX), cell cycle genes, ROS production, homeobox genes, immune genes, collagen formation genes and genes involved with cell structure, communication, adhesion and migration [55,62,73,74,76,77,78,79].

The observation that the Wnt and MAPK/Erk pathways are ubiquitously expressed in regeneration has been noted by other studies [62,76], in particular, Cary et al., (2019) found that early responses to regeneration are the most similar across species and that for species that can undergo WBR, there may be a conserved set of genes that have a similar temporal expression profile. This is not overly surprising as both pathways are highly conserved and both function in processes that are universally required in animals for development across multiple phyla [80,81,82].

Orphan genes also have a particularly important focus in regeneration, due to their ability to explain why some highly specialized morphologies and traits evolve in a lineage-specific manner [83]. A notable example of this comes from one of the major vertebrate models for regeneration, the salamander (Order Urodela), which is one of the few vertebrate groups containing members that can regenerate large portions of their body plans [49]. The salamander’s proficiency for regeneration relies on the recruitment of an orphan gene called *Prod1* (identified in *Ambystoma* spp. and *Notophthalmus viridescens*), which integrates into evolutionarily conserved vertebrate tissue repair and wound healing pathways in order to initiate a regenerative response [84,85,86,87]. This initiation and early recruitment of novel genes during regeneration is mirrored in *Hydra*; it was shown that cnidarian-specific novel genes (15/382 novel genes identified were active during regeneration) are recruited in the early stages of regeneration and conserved genes are recruited later in the response [66]. Interestingly, in salamanders, while the orphan gene is clearly implicated in the initiation of regeneration, in order to proceed it recruits conserved metazoan pathways [87]. This recruitment of conserved genes into lineage-specific processes has also been suggested to be mirrored in cnidarians in other processes outside of regeneration, such as toxin gene formation [31], novel morphologies [88] and other novel processes [89]. Although a tempting solution, orphan genes alone cannot explain the complexity and evolution of novel functions in taxa and it is likely regeneratively-competent organisms use a combination of lineage-specific and conserved genes to initiate and complete regeneration.

## 3. Overview of Sea Anemone Molecular Regeneration Studies

Few studies have investigated the functional genomic response of sea anemones to catastrophic damage that results in WBR. To date, three key studies have examined the molecular response in the model *N. vectensis*, each addressing different aspects of regeneration. DuBuc et al., (2014) used microarray analysis, qPCR and in situ hybridization to assay both wound healing and WBR following oral-aboral bisection, finding that many genes required for healing a puncture wound are also activated during transverse WBR [63]. Warner et al., (2018) produced a comprehensive transcriptomic data set from 6 week juvenile anemones (–1 (uncut), 0, 2, 4, 8, 12, 16, 20, 24, 36, 48, 60, 72, 96, 120 and 144 hpa (hours post-amputation)) for comparative analysis of regeneration and development, which can be mined in their online tool NvERTx (http://nvertx.ircan.org/ER/ER_plotter/home, accessed on 30 May 2021) [90]. This data is further explored in a pre-print released by the authors on bioRxiv [75] and is discussed here in more detail under the subheading ‘Is regeneration a recapitulation of development?’ Finally, Schaffer et al., (2016) have examined the transcriptomic response in adult anemones (4 months) in both the head and physa at multiple timepoints (0, 8, 24 and 72 hpa). This study found that each half followed a similar transcriptomic trajectory, with the latest timepoint (72 hpa) post-dissection showing the most dissimilar transcriptional response between the two regenerating halves [73].

**Figure 1:**
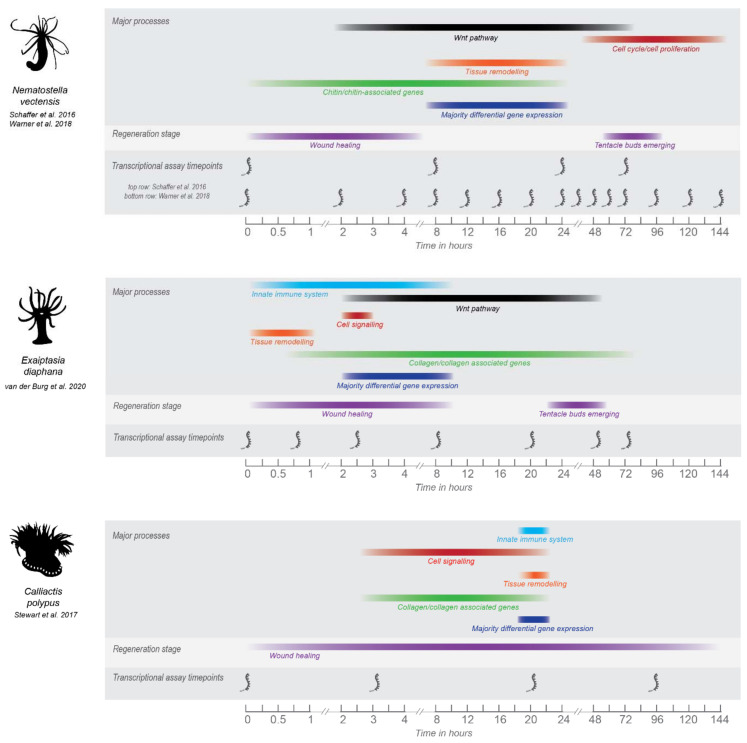
Overview of the major processes in regeneration as determined by transcriptomic studies performed in *N. vectensis, E. diaphana* and *C. polypus*. Major processes shown were chosen based on those highlighted by the authors in each study. Timepoints for which major processes are shown are based on either when the majority of differential gene expression is occurring for that process, or when significant gene ontology patterns are occurring. For visual ease, processes are shown as colored bars that span the timepoints for which gene expression was significantly detected. However, the gene expression occurring at timepoints between transcriptional data sets is unknown and so this should be considered a general overview only. Gene expression for each process may also be occurring at other timepoints in the regeneration datasets for each species, colored bars cover the timepoints for which significant expression or differential expression was occurring. Some of the major morphological processes are shown for context, timepoints for which are taken from the respective studies. For *C. polypus* and *E. diaphana,* timing of wound healing and regeneration need further refining and are based on visual evidence only. *Nematostella* wound healing and regeneration times are well-characterized on a cellular/morphological level. See Supplementary File S1, Table S1 for image copyrights and attributions and Table S2 for further information and source of data for this figure.

In other sea anemones, one study in *C. polypus* has examined the transcriptomic response over time (0, 3, 20, and 96 hpa) to longitudinal dissection of individuals that were quartered [78] and one study in *E. diaphana* has examined the transcriptomic response of head regeneration (from the aboral portion) over time (0, 45 min, 2.5, 8, 20, 48 and 72 hpa) following transverse dissection [91]. An overview of the timing of major gene expression processes that occur during regeneration in these three sea anemones is demonstrated by Figure 1. Virtually no molecular studies of regeneration have been performed in sea anemones outside of these three organisms. Further, each study has used different experimental strategies to assay regeneration, and hence, not all aspects of regeneration can be comprehensively and equivocally compared.

**Figure 2:**
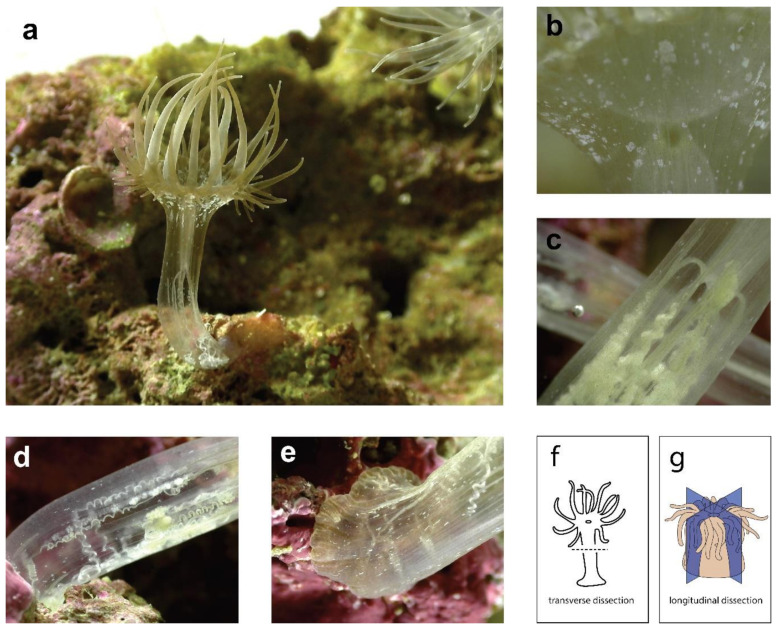
Multiple images of a bleached *E. diaphana* individual, showing the internal tissue organization. (**a**) an *E. diaphana* individual attached to a coral rock, in an upright position; (**b**) a portion of the oral disc showing where the mouth meets the pharynx; (**c**) the middle of the body column where the mesentery filaments are attached to the end of the pharynx (enterostome); (**d**) lower half of the body column, showing mesenteries and acontia; (**e**) lowest portion of body column and pedal disc (**f**) diagram of sea anemone showing transverse dissection plane; (**g**) diagram of sea anemone showing longitudinal dissection planes, reproduced with permission from [78]. See Supplementary File S1 for image copyrights and attributions.

A major difference between these studies is the plane of dissection. In *Nematostella* and *E. diaphana*, individuals were dissected once at a perpendicular angle to the oral-aboral axis (or ‘transversely’) to create two halves. In *C. polypus*, individuals were dissected into quarters through the oral-aboral axis (or ‘longitudinally’) and quarters were able to be used as replicates, as each contains approximately the same tissues and cell types. Differences in regenerative ability and the genetic signature of regeneration in ‘transverse’ vs. ‘longitudinal’ halves should be expected (and were observed in these studies) due to the different tissues and structures that must be regenerated. For example, transversely dissected halves must regenerate either an entirely new crown or pedal disc, whereas portions of both these tissues are still present in longitudinally dissected halves. Transversely dissected sea anemones may also show differences in regeneration depending on the specific location of the dissection e.g., through or below the pharynx. Oral and aboral halves of *Nematostella* (dissected below the pharynx) show similar trajectories in terms of both the transcriptional response and the time required to regenerate [73]. Anecdotally, in personal observations of *E. diaphana,* the ability of oral halves to regenerate shows major differences depending on where they are cut. Oral halves cut through the pharynx are ‘donut’ shaped and do not appear able to regenerate. Further formal investigation of this phenomenon is warranted, and in particular, investigation of the similarity of the molecular strategies employed by regenerating oral and aboral halves in *E. diaphana*. To demonstrate the tissue organization of sea anemones, Figure 2 shows images of an *E. diaphana* individual, through which the internal structures can be easily observed. The dissection planes are also illustrated.

The expression of the Wnt pathway is a key aspect of regeneration. The main gene expression difference observed between *C. polypus* and the other sea anemones (i.e., *Nematostella* and *E. diaphana*) is the absence of Wnt signaling in *C. polypus* (Figure 1 and discussed by [91]). This is not unexpected, as the primary role of the Wnt pathway is to establish longitudinal axis polarity, and in the *C. polypus* study the oral and aboral tissues were retained in each dissected quarter. In *Nematostella* and *E. diaphana* Wnt signaling is found throughout the time course, with significant differential expression occurring primarily in the ‘middle’ timepoints (Figure 1). In *E. diaphana,* differentially expressed Wnt pathway genes were only upregulated, and upregulation occurred at timepoints 2.5, 8, 20 and 48 hpa (see Figure 5 of [91]). Notably, this includes genes that are also negative regulators of the Wnt pathway (*notum1a*, *sFRP-3/FzB, DKK*), which are expressed throughout these timepoints, so the expression pattern may be more complex than it initially appears. In *Nematostella,* Wnt pathway genes are highly polarized across the oral-aboral axis i.e., major differences in expression are seen in oral vs. aboral regenerating halves [73]. As only oral-regenerating halves were assayed in *E. diaphana*, only the expression of this half can be compared to *Nematostella*.

Schaffer et al., (2016) identified three major Wnt clusters in oral regenerating halves of *Nematostella,* which were early-upregulated (largest cluster of transcripts), down and back up, and late-upregulated. One of these genes, *Wnt5*, is highlighted by the authors as a potential driver of tentacle evagination, which is a well-characterized mechanism in *Hydra* [92]. This gene is not DE in *E. diaphana* regeneration (*Wnt-5b-like* is annotated in the *Exaiptasia* genome). It is possible that the statistical power to detect differential expression of Wnt pathway genes is lost in this *E. diaphana* study, as the entire ‘body’ portion of tissue was used for RNAseq, versus in *Nematostella* which used 1 mm wide dissected pieces of tissue from the regenerating side [73]. This is particularly important for Wnt genes, as a study in *Hydra* [92] shows that highly localized gene expression of *wnt5*, *wnt8* and *frizzled2 (fz2*) occurs at the base and tips of regenerating tentacles. Surprisingly, no studies have thoroughly examined tissue localized gene expression of Wnt genes in this way (e.g., through protein detection methods such as Western blot or immunostaining, or through mRNA expression detection methods such as in situ hybridization) during regeneration in *Nematostella*, although this has been performed during embryogenesis [93]. Trevino et al., (2011) showed *β-catenin* is involved in oral regeneration in *Nematostella*, by inducing ectopic head regeneration in aborally regenerating individuals (i.e., a head grew where a physa should have). However, this study only used qPCR to investigate gene expression [94]. Oddly, Schaffer et al., (2016) did not find DE of *β-catenin* in their transcriptome study, and it was also not found to be DE in *E. diaphana* by van der Burg et al., (2020). This gene does show an early spike in expression in *Nematostella* using the NvERTx plotter tool ([90] and see Supplementary Table S3 for data), which is consistent with the pattern seen in the Trevino et al., (2011) qPCR results.

Lastly, the Wnt secretion regulator *WLS* (*Wntless*) and the Wnt inhibitors *notum1a*, *DKK* (Dickkopf) and frizzled related genes *sFRP/FZb* and *sFRP1* (secreted frizzled related proteins/frizzled) all show similar patterns of expression in *Nematostella* and *E. diaphana.* Schaffer et al., (2016) [73] classified all of these as part of the ‘early-upregulated’ genes (where 8 hpa is the earliest timepoint assayed). In *E. diaphana,* these genes are also expressed at 8 hpa or earlier (as indicated in Figure 5 of van der Burg et al., (2020) [91]) as follows: *Notum1a* (2.5, 8, 20 hpa), *WLS* (2.5, 8 hpa), *DKK3* (8 hpa), and frizzled related genes *sFRP1* and *sFRP3/FZb* (2.5, 8, 20, 48 hpa). Further evidence from the NvERTx plotter tool [90] indicates that *WLS* and *notum1a* genes are also being expressed even earlier than 8 hpa in *Nematostella* (see Supplementary Table S3 for data), similar to *E. diaphana.* The expression pattern of these genes during regeneration in both sea anemones (which are typically being expressed early and are sustained, for further evidence in *Nematostella* see Additional File S9: Figure S4 of [73]), are interestingly more comparable to the expression pattern in Planaria, than the cnidarian *Hydra*. The relationship between Wnt gene expression in *Nematostella* vs. Planaria vs. *Hydra* is explored by [73]. While some genes show polarized expression between *Nematostella* vs. Planaria, notably the major drivers of regeneration in *Hydra*, *Wnt3* and *β*-catenin [68], are missing from Nematostella (and *E. diaphana*) regeneration programs. Further conclusions on the similarity of expression between the two sea anemone species and other regenerative models (e.g., *Hydra* and Planaria) requires more fine-scale gene and protein expression assays throughout the tissue. Further proof-of-function assays are also required for Wnt pathway genes, for example through RNAi/gene silencing of Wnt components, or in the case of sea anemones outside *Nematostella*, testing ectopic oral regeneration activation through treating regenerating individuals with the Wnt pathway/ β-catenin activator alsterpaullone [94].

Another major difference between the regenerative response in *C. polypus* vs. both *Nematostella* and *E. diaphana* is the timescale. Regeneration completes quicker in *Nematostella* and *E. diaphana*, roughly completing within 72–96 h (3–4 days), at which point wound healing and regeneration still appears to be completing in *C. polypus.* However, given the major difference in dissection in *C. polypus,* comparing anatomical timescales to the other two sea anemones is largely uninformative. Stewart et al., (2017) observed that dissected quarters required approximately 16 days to suture their bodies into closed forms again. Notably, transcriptionally *C*. *polypus* has returned to baseline levels by the last timepoint (96 hpa), which is a pattern that holds true for the other two sea anemones. This indicates regeneration in sea anemones is primarily characterized by an early burst of transcriptional activity, which quickly returns to baseline, and has likely set-in motion the gene expression required to continue wound healing and regeneration of the individual. Future studies in sea anemones should combine transcriptomic and proteomic approaches to further tease apart this response, such as a study in *Hydra* which combined and compared both molecular responses [66]. Proteomic activity frequently shows a different (delayed) timeline following the initial burst of transcription and may reveal regulation missed by transcriptomics.

## 4. Ecological, Evolutionary and Genomic Context of Sea Anemone Regeneration

**Figure 3:**
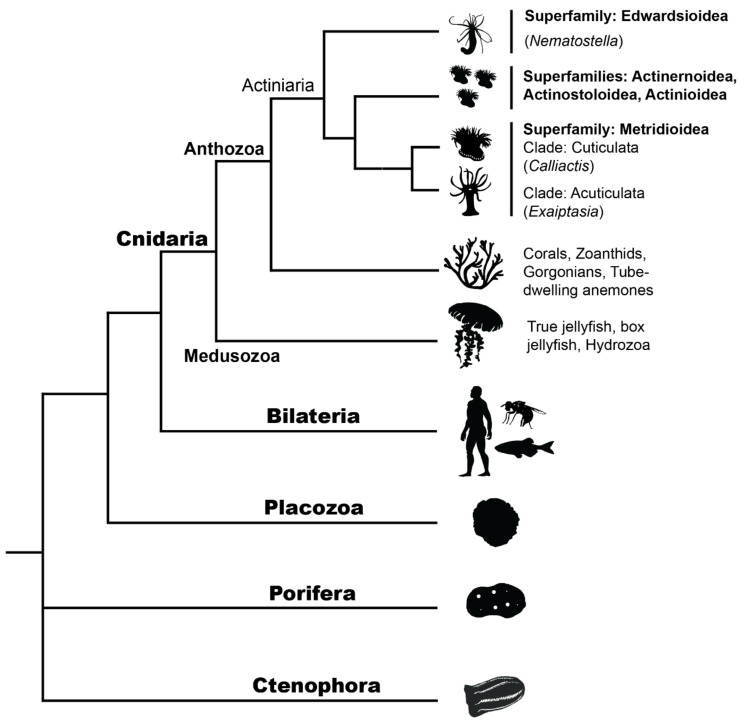
Metazoan phylogeny shows the phylogenetic placement of Cnidarians, including the two major classes within Cnidaria: Medusozoa (e.g., jellyfish, hydrozoans) and Anthozoa (e.g., corals, sea anemones). All non-actiniarian Anthozoans are shown as a single branch for visual simplicity. Internal branches within the order Actiniaria show where *Nematostella, Exaiptasia* and *Calliactis* species sit within the superfamilies Edwardsioidea and Metridioidea. Actiniarian branches have been simplified here for visual purposes; however, Actiniaria is not monophyletic and there is conflict over current hierarchies, see [21]. See Supplementary File S1, Table S1 for image copyrights and attributions.

Relatively few whole-body regeneration studies at a molecular level have been performed on cnidarian species, and even fewer on sea anemones. Some of these in cnidarians (outside of sea anemones) include *Hydra* (Medusozoa: Hydrozoa) (e.g., [66,68,71]), *Hydractinia echinata* (Medusozoa: Hydrozoa) (e.g., [58,70]), *Aurelia aurita* (Medusozoa: Scyphozoa) [95], and with most regeneration studies on corals (Anthozoa: Scleractinia) pertaining to wound or lesion repair rather than WBR (e.g., *Acropora aspera* [96] and *Montastraea cavernosa* [97]). The phylogenetic position of major classes within Cnidaria and the position of Cnidaria relative to other metazoans is illustrated in Figure 3.

### 4.1. Linking Life History Strategies and Regeneration

Unusual mechanisms of development, reproduction and regeneration have been observed in cnidarians and the line between what constitutes each of these processes is not always easily separable or definable. Examples of these mechanisms in Cnidaria include: the ‘reverse development’ mechanism in the immortal jellyfish *Turritopsis dohrnii* (Medusozoa: Hydrozoa) [98], the ‘budding’ reproduction mechanism and the ‘morphallaxis’ and ‘reaggregation’ regeneration mechanisms in *Hydra* [68,99,100,101,102,103], the ‘multiple-crown/multiple-physa’ mechanisms in *Nematostella* [104] and the ‘resymmetrization’ mechanism observed in the moon jellyfish ephyra *A. aurita* [95]. Interestingly, very few sea anemones outside of *Nematostella* have been observed with multiple ‘crowns’ (a process also referred to as heteromorphosis), but this is inducible in some sea anemones by introducing a small transverse incision of sufficient depth into the body column (e.g., in *Harenactis* sp. [45], and pers. observ. of *Actinia tenebrosa*), which may or may not result in complete transverse fission in some species (discussed by [105]). This mechanism illustrates an interesting overlap between regeneration and development strategies in sea anemones, and further highlights the diverse evolutionary and life history strategies that occur in phylum Cnidaria.

The evolution of regeneration in sea anemones is linked both to their life history strategy, ecology and to their phylogenetic position. *Calliactis* and *Exaiptasia* are phylogenetically closer (see Figure 3) and may for this reason be expected to be more similar, but *Nematostella* and *Exaiptasia* share more similar life history strategies e.g., both frequently propagate asexually through either fission or pedal laceration [34,104] and morphologically are more similar i.e., much less dense tissue than *Calliactis*. *Exaiptasia* and *Nematostella* both display clonal colony distributions on global scales [106,107], owing in part to their ability to proficiently reproduce asexually. Importantly, *Calliactis* species can reproduce naturally by longitudinal fission [108], but this is thought to occur infrequently in comparison to sexual reproduction. No studies of *Calliactis* on a population scale have been undertaken that give further insight into its global distribution and its preferred modes of reproduction. With these considerations in mind, it is expected that *C. polypus* and *E. diaphana* might share more similar gene sets used during regeneration, which in part appears to be true (see ‘*Regeneration geneset evolution*’). However, the evolution of the *C. polypus* regeneration gene set was not directly compared to the gene set from *E. diaphana*, and the difference in dissection methods means that only broad inferences should be made. Further, it would be expected that *Nematostella* and *E. diaphana* would morphologically and temporally be more similar in their modes of regeneration (i.e., stages and timing of morphological events, such as pharynx formation or tentacle bud emergence). Video observation of *E. diaphana* [91] indicates the overall timing may be quite similar to *Nematostella*, but specific cellular and tissue regeneration stages have not yet been characterized in *E. diaphana* as they have been in *Nematostella* [109,110].

### 4.2. Genomic Resources for Actiniaria

The importance of increasing genomic resources to answer evolutionary questions is particularly essential when investigating gene sets across the long timescales that separate species from different Actiniarian families (i.e., up to 400–600 mya [22]). Few genomes currently exist for sea anemones; these include *Nematostella vectensis* (Edwardsiidae) [6], *E. diaphana* (Aiptasiidae) [111], *A. tenebrosa* (Actiniidae) [112], *Anemonia viridis* (Actiniidae) [113] and *Actinia equina* (Actiniidae) [114]. Multiple assemblies have recently been made available on the NCBI genome repository, but lack an associated publication, including *Heteractis crispa* (ASM1516403v1), *Heteractis magnifica* (ASM1176337v1), *Stichodactyla helianthus* (ASM1516394v), *Stichodactyla mertensii* (ASM1180000v1) and *Phymanthus crucifer* (ASM985815v1). Currently, all genomes for sea anemones are at scaffold or contig level assembly (no chromosomal level assemblies) and only one assembly (*A. equina* [114]) has utilized long read technologies such as PacBio SMRT sequencing. Further genomic resources are needed to more fully understand how novel, conserved and ancient genes are co-opted in Actiniaria, and how this pattern contributes to complex processes such as regeneration.

### 4.3. Regeneration Gene Set Evolution

In Actiniaria, both ‘old’ and ‘new’ genes contribute to the molecular profile of regeneration. This is demonstrated by a gene family evolution analysis of differentially expressed genes during regeneration in *E. diaphana* across cnidarian and metazoan species [91]. Specifically, Figures 7 and 8 of van der Burg et al., (2020) illustrate the number of regeneration dataset orthologs present in cnidarian and metazoan species, and how these genes have been gained or lost on the evolutionary tree. In general, the number of orthologs present in each species is explained by phylogenetic distance from *E. diaphana* i.e., the closer a species to *E. diaphana* phylogenetically, the more orthologs it has in common with the regeneration dataset. However, while it would initially appear that the majority of genes used in *E. diaphana* regeneration are actiniarian-specific (Figure 7 of [91]), the majority (~70%) actually evolved in the common ancestor of Cnidaria and Bilateria (Figure 8 of [91]). This is not to say there are no cnidarian-specific innovations, as >14% of the regeneration dataset evolved in the Anthozoan common ancestor, and approximately 7% evolved in the Actiniarian common ancestor. This study has primarily used reference transcriptome datasets from sea anemones and other metazoan species to explore the evolution (gene gain/loss) of the regeneration gene set in *E. diaphana.* While this presents some interesting general insights into the evolution of regeneration in sea anemones, this approach has limitations. Gene counts likely need refining and the paucity of genomic datasets across large evolutionary distances makes it difficult to make any strong inferences about when any genetic signatures of regeneration may have evolved or been lost. In particular, generating more complete genomic assemblies for sea anemones will give more confidence in detecting orphan genes in sea anemone taxa and their putative role in regeneration.

### 4.4. Broad-Scale Evolution of Regeneration

Studies in other sea anemone species have not investigated the evolution of the regeneration dataset in such a broad sense, but comparisons to other regeneratively competent organisms have been made. Warner et al., (2019) [75] present an investigation into the evolution of the *Nematostella* regeneration dataset in the context of embryogenesis, but this study does not further investigate the broader evolutionary context of these genes, as their primary question was to compare to what extent regeneration in *Nematostella* is a recapitulation of embryogenesis. In fact, to a large extent the evolutionary context of the *Nematostella* regeneration dataset has not been investigated. Other key studies in *Nematostella* regeneration use either a primarily cellular approach [110], or have used molecular techniques to understand the fine-scale differences and similarities between wound healing and regeneration [63]. Schaffer et al., (2016) [73] comprehensively compare head vs. physa/tail gene expression in *Nematostella* and Planaria and interestingly find that many highly conserved genes such as *HOX* genes, Wnt pathway genes (discussed in an earlier section here) and transcription factors that are expressed during regeneration in both models, show both concurring (expressed in the same axis in both organisms) and polarized (expressed in opposite axes in each organism) expression. This observation is interesting considering these genes families have highly conserved roles in driving body plan morphology and orientation, but may somewhat be attributed to the major structural and morphological differences between sea anemones and planarians. To what extent the *Nematostella* and *C. polypus* regeneration datasets may be novel or conserved in comparison to other Cnidarians (or other taxa) has yet to be explored. With the transcriptomic datasets available for *Nematostella* [73,75,90], and with the recent release of improved gene models [115], the gain/loss and evolution of genes involved in regeneration could be comprehensively explored for this species and how novel the gene set is could be elucidated.

### 4.5. Regeneration and the Immune System

Regeneration and the immune system are intrinsically linked, as any wounding or damage sufficient to result in regeneration will also expose the organism to environmental pathogens requiring an immune response [116,117]. In general, the evolution of a more complex and adaptive immune system has been correlated with a decrease in regenerative capability, although how this varies across phylogenetically distinct taxa has yet to be comprehensively explored [15,116,117,118,119,120,121]. A few studies have suggested that an early and tightly-controlled inflammatory/immune response during regeneration may be one of the few responses shared across the animal kingdom [76,122,123], although studies provide conflicting evidence for whether components of the innate immune system can positively (e.g., in coral wound healing [96] and in a study on *Hydra* and *Schmidtea* [121]) or negatively (e.g., in *Xenopus* [74,119]) affect regeneration.

Several studies have characterized the immune gene repertoire in cnidarians and have shown a high degree of conservation of innate immune genes across many representatives of phylum Cnidaria and has provided insights into their evolution in metazoans [6,19,91,111,124,125,126,127,128,129]. As well as a core set of highly conserved immune genes, a common thread in the study of cnidarians is the identification of a rich novel immune gene repertoire [19,20,126,129,130], and although this provides an initial insight into the novel molecular tool kit of these organisms, few functional analyses of these genes currently exist. Investigation of the innate immune system can provide two important perspectives on regeneration: how conserved genes may be co-opted into a lineage-specific function, and how novel genes may initiate or contribute to the lineage-specificity of regeneration. Although it is outside the scope of this review to examine the literature on functional analyses of innate immune genes in sea anemones and cnidarians, we point the readers to some interesting work on immune gene function in *Nematostella* (e.g., nematosomes are part of the immune system and are characterized by lineage-specific genes [88] and NF-kB and TLR are required/expressed during embryonic development [131,132]).

In *C. polypus, E. diaphana* and *Nematostella* there is little congruence between the specific immune genes that are expressed during regeneration. The ‘early-restricted’ pattern of immune expression holds true for *E. diaphana*, as no innate immune genes are differentially expressed at any time point after 8 hpa. One exception to this is the downregulation of one novel putative immune gene (TIR containing) at 48 hpa. Therefore, no evidence is presented that suggests the innate immune system impedes regeneration to a large extent in *E. diaphana,* as immune expression is maintained at a baseline level throughout each of the later timepoints. Several immune genes are differentially expressed in *C. polypus,* but few are the same as in *E. diaphana*, with the only shared features being an Interleukin-1 like receptor gene and genes containing the domain ‘Scavenger receptor cysteine rich’. Immune genes in *C. polypus* are predominantly expressed at 20 hpa, which the authors classify as a late response, and is later than innate immune genes are expressed in *E. diaphana*. Morphologically, *C. polypus* appears to be engaged in wound healing and regeneration throughout the entire transcriptional time course, but gene expression indicates that the stress and wound healing response mostly occurs at the earliest time point (3 hpa) [78]. The authors do not comment specifically on what governs wound healing after 3 hpa, however, they do postulate that a switch from transcriptomic to proteomic activity between 20–96 hpa may explain the return to baseline of all transcript expression observed at 96 hpa. The role of the innate immune system during regeneration in *Nematostella* has not been explored in the RNAseq studies performed by Warner et al., (2019) and Schaffer et al., (2016) [73,75]. DuBuc et al., (2014) does investigate this (using microarray analysis and antibody based detection), insofar as the study finds that the MAPK/Erk pathway, which has a role in immune regulation, is crucial for both wound healing and regeneration [63].

The relationship and interplay between regeneration and the immune system requires further exploration in sea anemones. From a genomic perspective, investigating whether knocking out the expression of components of the innate immune system would impede, inhibit, benefit, or have no effect on regeneration would give an interesting insight into this relationship. From a cellular perspective, the role of specialized cells and/or tissues, e.g., mesenteric filaments, have yet to be comprehensively explored. In *Nematostella*, mesentery interact with the wound site during regeneration (in both oral and aboral wounding) and their behavior can be used as a landmark for stages of regeneration [63,109,110]. *Nematostella* mesenteric filaments show diverse expression of proteolytic genes (in particular trypsin family genes) and they may have a substantial role in the innate immune response and in tissue remodeling [133]. Some localized gene expression has been explored in the mesenteries (see [63], in particular Figure 6) and video observation of regeneration in *E. diaphana* (see Video 1 of [91]) and in *C. polypus* (images in the supplementary information [78], and observations relayed through pers. comm.) indicate there is substantial activity of the mesenteries during regeneration. Mesenterial filaments can be observed moving rapidly both outside of the body and within the body column during regeneration in *E. diaphana* in particular and especially at the early stages of regeneration. Together, these observations and studies indicate mesenterial filaments play a key role in wound repair and regeneration. Given the high enzymatic activity and high mobility of this tissue, we suggest perhaps this role is achieved through two key functions: 1. To degrade or ‘clean-up’ cellular debris at the damage site, thereby aiding in tissue remodeling; and 2. To defend against invading pathogens through enzymatic degradation and thereby aid in innate immune function.

### 4.6. Is Regeneration a Recapitulation of Development?

Regeneration and development are famously intertwined and the relationship between the two processes has been investigated for more than a century [99]. Many studies and reviews in the fields of both embryonic developmental biology and regeneration biology have contemplated the extent to which regeneration is a recapitulation of development, with the general consensus that it is likely driven by the same gene networks, but the specific pathways deployed may be regeneration-specific [15,48,134]. In part, this question has been difficult to answer due to a lack of large-scale comparative analyses with genomic datasets that sample comprehensively across both developmental and regenerative time courses. In *Nematostella,* Warner et al., (2019) found a “partial redeployment” of the embryonic gene network is observed during regeneration [75,90]. This study identified key embryonic modules that are activated during regeneration to achieve basic cellular functions and also detected a regeneration-specific signature of expression driving the response. A subset of the regeneration-specific genes (48/124) were identified as transcriptionally silent until regeneration is activated. Further, this study identifies a significant contribution from apoptosis and apoptotic signaling in regeneration, which are key drivers of Wnt signaling. This has also been identified in *Hydra* [68] (although some of the specific Wnt pathway genes expressed are different, see section ‘Overview of sea anemone molecular regeneration’). Other studies in acorn worm, Axolotl and *Polypterus* (fish) show similar results, although different extents of recapitulation are noted [55,135].

In the future, it will be interesting to see further research on the specific evolution of regeneration modules and how conserved or unique these are across taxa. A theoretical evo-devo framework was proposed by Tiozzo and Copley (2015) to explain a potential evolutionary history of regeneration across taxa. They propose a modular framework where a regeneratively competent ancestor may possess a robust or ‘canalized’ module (gene set or toolkit that is resistant to perturbation) and a more ‘plastic’ module, which together are required to confer a high regenerative capability. These modules are passed down to descendent lineages, where the robust module remains unchanged, but the plastic module changes in response to environmental factors and selection pressure. The result is different regenerative capabilities in each lineage, but both modules are required for regeneration [15]. Such a framework could potentially be used to describe embryonic development as a canalized module, and regeneration as a plastic module, which could wholly or in part have evolved from a module initially activated in development, and subsequently diverged to become a regeneration-specific module in competent taxa. This framework of ‘emergence from development’ could partly explain the most conserved molecular feature of regeneration—the early expression of Wnt and MAPK/Erk pathways. In fact, early developmental pathways tend to be the most conserved across phyla, where the ‘mid-developmental transition’ period is the most divergent within a phyla i.e., is associated with more species-specific suites of genes [81].

In the context of sea anemones, few transcriptomic developmental datasets currently exist, and all datasets are specific to Nematostella [75,90,104]. While E. diaphana is described as a ‘model’ species in the context of coral symbiosis [33,136,137,138], and has a sequenced genome [111], a major barrier to establishing this species as a model for other research areas is that (to the best of our knowledge) no laboratories have been successful in achieving ‘settling’ of the planula stage [139]. The inability to settle the planula is a major barrier to generating a complete developmental time course dataset for E. diaphana and therefore, it cannot be used to test to what extent regeneration is a recapitulation of development. As a small observation, we note that the convergent co-module in regeneration and development identified by Warner et al., (2019) display some GO terms in common with the E. diaphana regeneration dataset [75]. This indicates that E. diaphana could follow a similar regenerative/developmental trajectory as Nematostella, that is, some recapitulation of development during regeneration could be occurring in E. diaphana. It is not unexpected that E. diaphana and Nematostella would have some similarity, as both essentially use regeneration as part of their reproductive repertoire. No developmental datasets exist for C. polypus, and whether it is possible to spawn and develop this sea anemone in the lab has not been examined.

Further insights into the extent that these processes overlap, both within a single species such as *E. diaphana* and in comparison to other sea anemones, will require substantial data generation and the establishment of robust methods for manipulating developmental stages in a lab setting.

### 4.7. Other Observations

Collagen has been identified as an important molecule in regeneration in *Hydra* (early expression is critical) [139], and increasing collagen expression during regeneration has been identified in *C. polypus* and *E. diaphana* [43,78,91], although the majority of expression occurs at later timepoints (see Figure 1). A putative novel collagen gene was also identified in *E. diaphana*, although this requires further validation. The role of collagen during regeneration is yet to be explored in sea anemones, but this could perhaps give some interesting insight into the tissue plasticity of sea anemones and how they achieve scar-free wound repair and regeneration. Additionally, *C. polypus* and *E. diaphana* have been observed to engage in a rhythmic peristatic muscle contraction or ‘pulsing motion’ during regeneration in [78,91]. Rhythm muscle contraction is the driving mechanism for regeneration in *A. aurita* ephyra [95] and is a feature of regeneration (dynamic circular contraction and expansion is observed at ~0–8 hpa) in *Nematostella* [132]. This ‘pulsing motion’ has not yet been investigated on a cellular or molecular level in sea anemones and whether this is a commonly observed phenomenon in Cnidaria is yet to be explored. In the future, other areas that should be explored more comprehensively in sea anemones are the mechanisms driving large-scale tissue rearrangement (i.e., tissue plasticity) and how different molecular components contribute to the mechanical aspects of regeneration.

### 4.8. Conclusions and Future Areas of Exploration

Studies on the molecular and genetic mechanisms underpinning regeneration in sea anemones are still in their infancy. So far, transcriptomic time course datasets have been generated for three sea anemone species (*N. vectensis*, *C. polypus* and *E. diaphana*), some evolutionary and phylogenetic analyses have been performed on regenerative gene sets (in particular for *E. diaphana*), and some evolutionary comparisons have been made to the programs of other organisms in the literature (e.g., *Nematostella* vs. Planaria). These studies have showed both conserved and novel components of regeneration, with some expected outcomes (e.g., sea anemones that show rapid regeneration responses tend to be characterized by an early burst in transcriptomic activity) and some unexpected outcomes (e.g., *Nematostella* and *E. diaphana* Wnt pathway expression appears more similar to Planaria than to *Hydra*). The next steps in research include: generating more comprehensive genomic and transcriptomic resources for sea anemones, generating developmental transcriptomic datasets, tissue-specific gene expression localization studies (e.g., ISH) and proof-of-function studies for target genes. 

## Data Availability

Not applicable.

## Supplementary Materials

The following are available online at https://www.mdpi.com/article/10.3390/genes12071072/s1, Supplementary File S1: Image copyrights and attributions and supporting data; Table S1: Image copyrights and attributions for black and white silhouettes, diagrammatic images and photographs used in this manuscript in all three figures; Table S2: Further information and source of data presented ‘Figure 1. Overview of the gene expression studies performed in Nematostella vectensis, Exaiptasia diaphana, and Calliactis polypus.’; Table S3: Wnt pathway gene expression timeline.
