# Supplementary material for: The Tentacular Spectacular: Evolution of Regeneration in Sea Anemones
Source: Genes (Basel). 2021 Jul 14;12(7):1072. doi: 10.3390/genes12071072 (PMC8306839; doi:10.3390/genes12071072)
Supplement: Supplementary file 1 [file genes-12-01072-s001.zip › genes-1180807-supplementary.pdf]

Supplementary File S1. Image Copyrights and Attributions and Supporting Data

The Tentacular Spectacular: Evolution of Regeneration in Sea Anemones

Chloé A. van der Burg * and Peter J. Prentis

School of Biology and Environmental Science, Faculty of Science, Queensland University of Technology,Brisbane 4000, Australia; p.prentis@qut.edu.au
* Correspondence: c.vanderburg@qut.edu.au

Table S1. Image copyrights and attributions for black and white silhouettes, diagrammatic images and photographs used in this manuscript in all three figures. All images have either been reproduced with explicit permission from the author/owner, or have been used in accordance with the copyright laws (i.e., links are provided here or images are attributed as public domain)

Species/organism	Type of image	Used in figure #	Attribution
Nematostella vectensis	Black and white silhouette	1, 3	Modified by Chloe van der Burg from https://www.flickr.com/photos/serc_biodiversity/30695685804/ Robert Aguilar. CC 3.0 Unported
Exaiptasia diaphana	Black and white silhouette Photographs	1,2,3	Chloe van der Burg
Calliactis polypus	Diagrammatic image	2	Reproduced with permission from Zachary Stewart. https://doi.org/10.1038/srep41458
Calliactis polypus	Black and white silhouette	1,3	Modified by Chloe van der Burg from http://phylopic.org/image/759759f0-a1c7-43b5-ac6b-df06a7f46a26/ Public domain dedication 1.0
Coral	Black and white silhouette	3	Chloe van der Burg

Jellyfish	Black and white silhouette	3	Modified by Chloe van der Burg from http://phylopic.org/image/defdbe58-28ab-4491-a43d-b88d1a8fa3bb/ Mali'o Kodis. CC 3.0 Unported
Human	Black and white silhouette	3	http://phylopic.org/image/c089caae-43ef-4e4e-bf26-973dd4cb65c5/ Public domain dedication 1.0
Fruit fly	Black and white silhouette	3	Image made by Jaye Newman, used here with permission.
Fish	Black and white silhouette	3	Public domain dedication 1.0
Placozoa (*Trichoplax*)	Black and white silhouette	3	http://phylopic.org/image/defdbe58-28ab-4491-a43d-b88d1a8fa3bb/ Mali'o Kodis. CC 3.0 Unported
Porifera (*Amphimedon queenslandica*)	Black and white silhouette	3	http://phylopic.org/image/8b28733a-9eab-4510-9dcf-9235aaf44d14/ Public domain dedication 1.0
Ctenophore	Black and white silhouette	3	Public domain dedication 1.0

Table S2: Further information and source of data presented 'Figure 1. Overview of the gene expression studies performed in *Nematostella vectensis, Exaiptasia diaphana,* and *Calliactis polypus*.' Processes and genes/pathways were chosen if they were identified as being significantly differentially expressed (DE) by the study, if patterns of gene expression showed significant gene ontologies associated with that process, or were otherwise identified as having an important role in regeneration for other reasons by the study.

**Process**<br>Criteria	***Nematostella vectensis***	***Exaiptasia diaphana***	***Calliactis polypus***
**Wnt pathway**<br>DE of genes in the Wnt pathway.<br>Gene ontologies associated with the Wnt pathway.	Differential expression of Wnt pathway components occur at many timepoints, however, the authors [1] describe that majority of transcripts show a peak early expression (8hpa) which typically lasts to 72hpa before dropping down (see qPCR data in Additional files of [1]). Further evidence from [2] (presented here in Table S3, and also see Figure 3 Di and Diii of [3]) indicates many of the transcripts identified as 'early-expressed' by [1] are being highly upregulated as early as 2hpa	All differentially expressed Wnt pathway genes are upregulated, at timepoints 2.5, 2.5, 8, 20 and 48 hpa. See Figure 5 of [4]	Not expressed (as expected due to nature of dissection method)
**Cell cycle/cell proliferation**<br>Gene ontologies associated with cell cycle and cell proliferation	[3] identifies regeneration module of genes associated with cell cycle and cell proliferation upregulated at 36-144 hpa (Figure 3 Ci, Cii, Ciii in study)	Not highlighted as major players in regeneration	Not highlighted as major players in regeneration
**Cell signalling**<br>Gene ontologies associated with cell signalling, response to signalling, signal receptor activity, signal transduction.<br>DE of genes associated with cell signalling.	Not highlighted by either study as being a major player in regeneration (as a caveat, this is a very generic term and more specific GO terms are examined instead)	Clusters of gene expression (showing significant GO enrichment with multiple terms related to cell signalling) show a peak in expression at 2.5hpa (Figure 3 of [4])	DE of genes associated with cell signalling occur at 3 and 20 hpa (Figure 5 of [5])

**Tissue remodelling**<br>DE of genes associated with tissue/ECM/cell remodelling.<br>GO enrichment of genes associated with cytoskeletal remodelling, cell movement.	DE of metalloproteinases (ADAMTS, astacins, other MPs) primarily upregulated at 8 hpa (qPCR data Additional File 6: Table S3 of [1]) and at 8 and 24 hpa (transcriptome data Additional File 5 : Table S4 of [1]) in the oral side.	Clusters of gene expression (showing significant GO enrichment with multiple terms related to cytoskeletal remodelling, cell movement) show a peak in expression at 0-45min (Figure 3 of [4])	DE of ECM remodelling genes 20hpa (Figure 5 of [5]).
**Chitin-associated genes**<br>DE of genes annotated as chitin.<br>DE of genes annotated as chitin binding or synthesising protein (e.g., GO:0008061: chitin binding).	[1] finds chitin/chitinase genes to be enriched, as well as chitin binding and secreting genes (see Figure 4 and in-text). qPCR shows majority of DE occurring at 0-24 hpa in oral regenerating halves.	Not DE in the timecourse (described in-text [4])	Not mentioned by study.
**Innate immune system**<br>DE of genes receiving annotation with 'GO:0002376 immune system process'.<br>DE of genes with immune related PFAM annotations<br>(e.g., TIR domain PF01582).	Not mentioned in either study [1,3]	Innate immune genes DE at 45mins, 2.5, 8 hpa. Novel immune genes also identified of potential importance (e.g, CniFL genes) see Figure 6 and Suppl. TS8 of [4]	Two Interleukin-1 receptor-like proteins identified as DE at 20hpa only (see Figure 5 of [5] and in-text).
**Collagen/collagen associated genes**<br>DE of genes annotated as collagens.<br>DE of genes known to increase collagen protein (i.e., *LARP*).	Not described by either study as having any substantial effects on regeneration [1,3]	Collagens are highlighted as potentially being important during regeneration and a novel collagen is identified. Genes annotated with 'Collagen' can be found DE at 2.5, 8, 20, 48 hpa and the collagen synthesising gene *LARP6* is upregulated at 45min, 2.5, 8 hpa (Suppl. TS5 of [4])	Collagens are highlighted as potentially being important during regeneration, *LARP6* is upregulated at 3 and 20 hpa (see Figure 5 of [5])

**Majority differential gene expression**	Figure 3c of [1] shows the majority occurs at 8 and 24 hpa.	Figure 4 of [4] shows the majority occurs at 2.5 and 8 hpa	Figure 4 of [5] shows the majority occurs at 20 hpa
**Wound healing** Morphological/visual evidence	[6,7] characterise this as complete by 6 hpa.	Visual evidence. Authors [4] describe this in-text and provide videos.	Visual evidence. Authors[5] describe this in-text and provide images in Suppl. file.
**Tentacle buds emerging** Morphological/visual evidence	[6] characterise this as occurring at Stage 2 on the NRSS, emerging at 60-96 hpa<br>[7] state true tentacle buds have formed at Stage 3, but appear in stage 2. (~2-5days)<br>All studies note timing is highly temperature dependant.	Visual evidence. Authors [4] describe this in-text and provide videos.	Not applicable, but full recircularization/ regeneration appears to take ~16 days (see Suppl. file)

Nematostella vectensis transcriptome studies [1–3]
Nematostella vectensis characterisation of wound healing and regeneration from a morphological and cellular perspective [6,7]
Exaiptasia diaphana transcriptome study [4]
Calliactis polypus transcriptome study [5]
(NB: There is a mistake in figure 5 of this paper. The authors generated transcriptomic data for 0, 3, 20 and 96 hpa, but have labelled the 3 hpa time as 4 hpa.)

Table S3: Wnt pathway gene expression timeline. Data is shown as 'Regeneration average counts (hours post amputation) -- log2'. Results are from searching the Warner *et al* 2018 [2] regeneration and embryonic data set plotter using key terms (e.g., 'wntless' or 'catenin' or 'notum'). UC = uncut.

Name	ID	UC	0h	2h	4h	8h	12h	16h	20h	24h	36h	48h	60h	72h	96h	120h	144h
Catenin beta	NvERTx.4.32434	7.8	7.87	8.43	8.75	8.48	8.68	8.93	8.98	8.78	8.54	8.39	8.23	8.24	8.14	8.07	7.92
Protein wntless homolog	NvERTx.4.10017	4.7	4.41	5.48	6.19	6.19	7.56	8.13	8.29	7.88	7.0	6.59	6.12	5.94	5.63	5.4	5.27
Notum1	NvERTx.4.158087	5.92	4.63	4.64	5.13	5.59	6.37	7.04	7.29	7.09	6.8	6.6	6.39	6.35	6.17	6.16	6.12

URL: http://nvertx.ircan.org/ER/ER_plotter/results [accessed 30-May-2021]

References

1. Schaffer, A.A.; Bazarsky, M.; Levy, K.; Chalifa-Caspi, V.; Gat, U. A Transcriptional Time-Course Analysis of Oral vs. Aboral Whole-Body Regeneration in the Sea Anemone Nematostella Vectensis. *BMC Genomics* **2016**, *17*, 718, doi:10.1186/s12864-016-3027-1.
2. Warner, J.F.; Guerlais, V.; Amiel, A.R.; Johnston, H.; Nedoncelle, K.; Röttinger, E. NvERTx: A Gene Expression Database to Compare Embryogenesis and Regeneration in the Sea Anemone Nematostella Vectensis. *Development* **2018**, *145*, dev162867, doi:10.1242/dev.162867.
3. Warner, J.F.; Amiel, A.R.; Johnston, H.; Röttinger, E. Regeneration Is a Partial Redeployment of the Embryonic Gene Network. *bioRxiv* **2019**, 658930, doi:10.1101/658930.
4. van der Burg, C.A.; Pavasovic, A.; Gilding, E.K.; Pelzer, E.S.; Surm, J.M.; Smith, H.L.; Walsh, T.P.; Prentis, P.J. The Rapid Regenerative Response of a Model Sea Anemone Species *Exaiptasia Pallida* Is Characterised by Tissue Plasticity and Highly Coordinated Cell Communication. *Mar. Biotechnol.* **2020**, *22*, 285–307, doi:10.1007/s10126-020-09951-w.
5. Stewart, Z.K.; Pavasovic, A.; Hock, D.H.; Prentis, P.J. Transcriptomic Investigation of Wound Healing and Regeneration in the Cnidarian *Calliactis Polypus*. *Sci. Rep.* **2017**, *7*, 41458, doi:10.1038/srep41458.
6. Amiel, A.R.; Johnston, H.T.; Nedoncelle, K.; Warner, J.F.; Ferreira, S.; Röttinger, E. Characterization of Morphological and Cellular Events Underlying Oral Regeneration in the Sea Anemone, Nematostella Vectensis. *Int. J. Mol. Sci.* **2015**, *16*, 28449–28471, doi:10.3390/ijms161226100.
7. Bossert, P.E.; Dunn, M.P.; Thomsen, G.H. A Staging System for the Regeneration of a Polyp from the Aboral Physa of the Anthozoan Cnidarian Nematostella Vectensis. *Dev. Dyn.* **2013**, *242*, 1320–1331, doi:10.1002/dvdy.24021.
